# Gap-plasmon-driven spin angular momentum selection of chiral metasurfaces for intensity-tunable metaholography working at visible frequencies

**DOI:** 10.1515/nanoph-2022-0075

**Published:** 2022-05-16

**Authors:** Younghwan Yang, Hongyoon Kim, Trevon Badloe, Junsuk Rho

**Affiliations:** Department of Mechanical Engineering, Pohang University of Science and Technology (POSTECH), Pohang 37673, Republic of Korea; Department of Chemical Engineering, Pohang University of Science and Technology (POSTECH), Pohang 37673, Republic of Korea; POSCO-POSCTECH-RIST Convergence Research Center for Flat Optics and Metaphotonics, Pohang 37673, Republic of Korea; National Institute of Nanomaterials Technology (NINT), Pohang 37673, Republic of Korea

**Keywords:** chiral plasmonics, chirality, metahologram, metasurface, tunable metasurface

## Abstract

Tunable metasurfaces can replace conventional bulky active optical modules to realize practical flat optical devices such as lenses, LiDAR, holography, and augmented reality. However, tunable metasurfaces have generally been limited to switching between two distinct states. Here, we present liquid crystal (LC) integrated chiral metasurfaces, of which the metahologram intensity can be adjusted continuously between fully ‘on’ and ‘off’ states. The chiral metasurface consists of a gap-shifted split ring resonator (SRR), and exhibits spin angular momentum selection that reflects left-circularly-polarized light but perfectly absorbs right-circularly-polarized light (99.9%). The gap-shifted SRR realizes spin angular momentum selection using a metal–dielectric–metal multilayer structure and thereby induces a strong gap-plasmonic response, achieving the maximum calculated circular dichroism in reflection (CDR) of 0.99 at the wavelength of 635 nm. With the chiral metasurface, metaholograms are demonstrated with tunable intensities using LCs that change the polarization state of the output light using an applied voltage. With the LC integrated chiral metasurfaces, 23 steps of polarization are demonstrated for the continuous tuning of the holographic image intensity, achieving measured CDR of 0.91. The proposed LC integrated spin-selective chiral metasurface provides a new resource for development of compact active optical modules with continuously-tunable intensity.

## 1 Introduction

Structured materials have been used to manipulate electromagnetic properties of light, such as amplitude, phase, polarization, and frequency [1], [2], [3], [4], [5], [6], [7], [8], [9], [10], [11], [12], [13], [14]. Recently, metasurfaces composed of two-dimensional (2D) materials that have arrayed structures at a subwavelength scale have emerged as ultrathin flat optical components [15], [16], [17], [18], [19], [20], [21], [22], [23], [24]. Structured materials can induce various optical responses that cannot be observed in nature depending on their periodicity, materials, and geometry of structures, therefore, the use of metasurfaces has been evaluated as a way to control light at will, including focusing light at the desired position [25], [26], [27], [28], displaying high-resolution holographic images [29], [30], [31], [32], [33], [34], [35], [36], miniaturizing sizes of high-brightness color pixels [37], [38], [39], tunable color display for sensing devices [40], [41], [42], and extreme beam-steering at desired angles with high efficiency [43]. Therefore, due to their compact form factor with subwavelength pixels, metasurfaces have the potential to be applied in augmented reality [44], LiDAR [45], photonic sensors [46, 47], dispersion controlling devices [48], selective light reflectors [49], [50], [51], [52], absorbers [53], [54], [55], [56], and microscopic components [57].

Tunable metasurfaces have been widely explored with chemical, thermal, and mechanical stimuli due to the requirement of real-time active modulation of optical responses at visible frequencies [21, 58, 59]. Various tunable materials, including phase change materials (PCMs) and liquid crystals (LCs), have been proposed to actively manipulate scattering properties with those stimuli [58, 60]. However, previous tunable metasurfaces have been mainly exploited for the active modulation between two or a few more particular states. For example, PCM metasurfaces including vanadium dioxide (VO_2_) [60], [61], [62], and germanium antimony tellurium alloy (GeSbTe, GST) [63, 64], controls the functionality of the metasurfaces using only two distinct states, neglecting the intermediate states. Additionally, in the case of LC integrated metasurfaces, even though the vectorial metaholography concept has been applied using LCs to increase the number of states, only LCP, RCP, and several elliptical polarization states is used, so the maximum number of reconstructed images is limited to nine [65]. Therefore, a method to fully utilize the elliptically polarized states between the RCP and LCP states of the tunable metasurface should be developed to increase the degree of freedom and for continuously tunable functionality.

Chiral metasurfaces are metasurfaces with structured materials that have mirror images that are not superimposable. Such metasurfaces can independently manipulate amplitude, phase, polarization, and frequencies of light depending on its spin angular momentum (SAM) [3, 5, 6]. Since independent control of different SAMs enables two selective scattering properties by manipulating spin states of input light, chiral metasurfaces have been actively investigated for tunable metasurfaces with polarization control of input light. Switchable images [66, 67], high-Q resonances [68], and nonreciprocal metaholograms [69] have been reported using chiral structured materials. However, previous chiral metasurfaces have been exploited only in the infrared regime [66, 67, 69], because strong chiral plasmonic responses require three-dimensional structures [3, 70], which cannot be fabricated using conventional methods that are compatible with complementary metal-oxide-semiconductor. Thus, the practical application of tunable chiral metasurfaces requires planar structured materials at visible wavelength scales with conventional fabrication methods.

**Figure 1:**
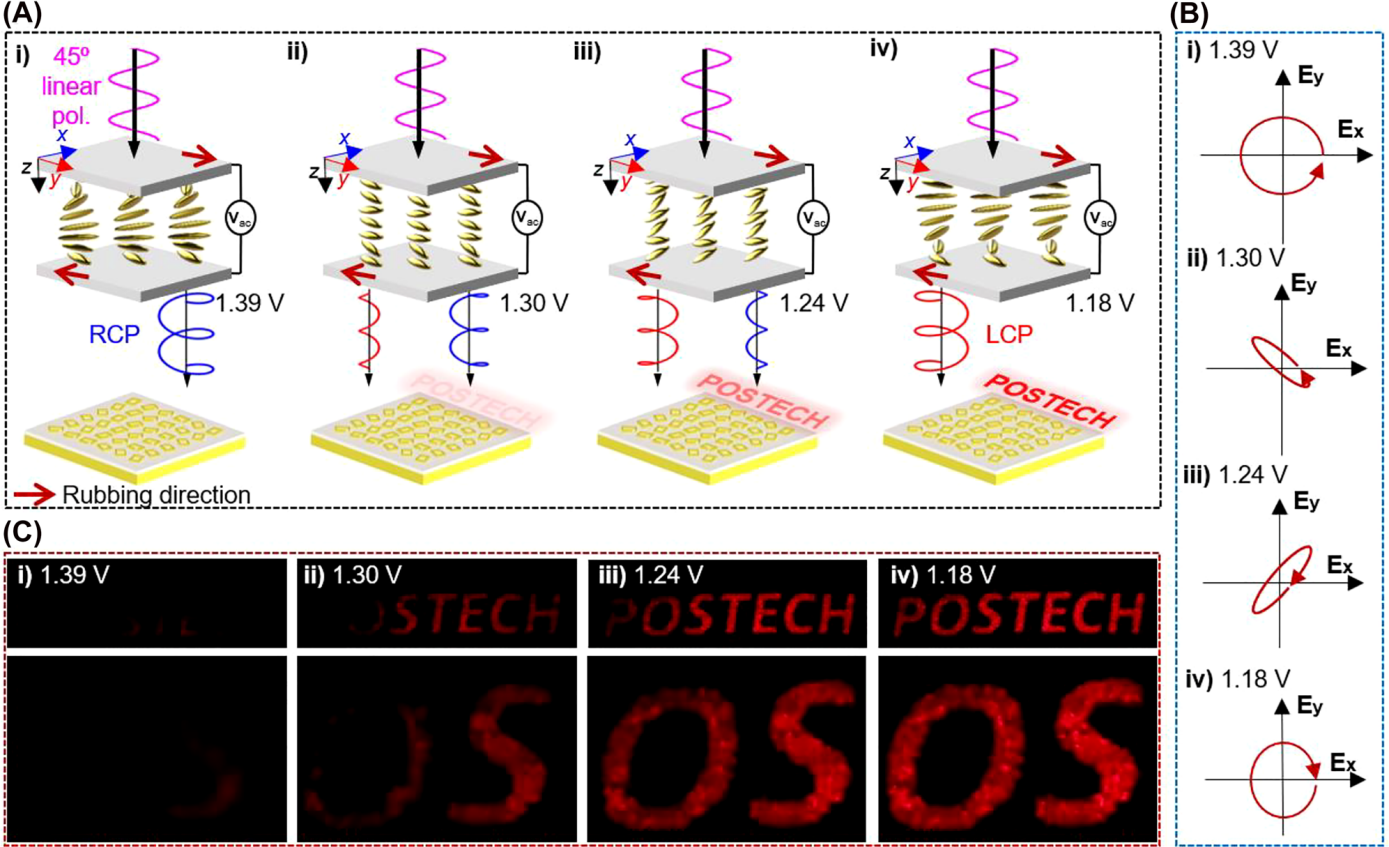
Schematic of electrically-tunable chiral metasurfaces for intensity manipulation of metaholography. (A) Schematic of electrically-tunable intensity of holograms with chiral metasurfaces. First, 45°-rotated linearly-polarized light is incident on the LC, and the polarization state of transmitted light is controlled by adjusting the applied voltage *V*_ac_. As *V*_ac_ is decreased from 1.39 to 1.18 V, the transmitted RCP gradually decreases, while the transmitted LCP increases. The chiral metasurface only display the holographic image under LCP, so the reconstructed hologram intensity can be continuously tuned by adjusting *V*_ac_. Red arrow: Rubbing direction. (B) Schematic of transmitted light polarization states from LC when *V*_ac_ was (i) 1.39 V, (ii) 1.30 V, (iii) 1.24 V, and (iv) 1.18 V. As *V*_ac_ decreased from 1.39 to 1.18 V, the polarization states changed from RCP to LCP. (C) Experimentally-obtained intensity-tunable holographic images when *V*_ac_ were (i) 1.39 V, (ii) 1.30 V, (iii) 1.24 V, and (iv) 1.18 V.

Here, an electrically intensity tunable metahologram is designed by integrating an LC with spin-selective chiral metasurfaces. The combination undergoes a continuous change of holographic intensity in response to the applied voltage *V*_ac_ (Figure 1). The arrangement of the LC is controlled by *V*_ac_, therefore enabling precise control of the polarization of the transmitted light. The transmitted light from the LC is left-circularly polarized (LCP) when *V*_ac_ = 1.18 V, and gradually becomes right-circularly polarized (RCP) as *V*_ac_ is increased from 1.18 to 1.39 V. The designed spin-selective chiral metasurface reflects incident LCP that creates images from an encoded computer-generated hologram, while perfectly absorbing the incident RCP. By exploiting the orthogonality of LCP and RCP, this LC-integrated spin-selective chiral metasurface can continuously control the intensity of a hologram by adjusting *V*_ac_ to change the proportion of incident LCP. Therefore, continuously intensity-tunable metaholography is achieved by controlling the voltage applied to the LC-assisted chiral metasurface.

## 2 Results and discussion

LC-integrated spin-selective chiral metasurfaces realize intensity-tunable metaholograms by adjusting *V*_ac_ (Figure 1A). Each LC functions as a quarter-wave plate with ordered molecules, and their optical axis is manipulated by changing the applied voltage. When 45° linearly-polarized light is incident to LCs, the output light can be changed to a desired polarization by adjusting *V*_ac_. At *V*_ac_ = 1.39 V, the output polarization of LC is RCP, and the output polarization state is converted to LCP at *V*_ac_ = 1.18 V (Figure 1B). In addition, at 1.18 V ≤ *V*_ac_ ≤ 1.39 V, the output polarization is elliptically polarized light, i.e., a combination of LCP and RCP. Thus, depending on the proportion of LCP in the output light, the SAM-selective chiral metasurfaces produce holographic images with different intensities (Figure 1A–C). When *V*_ac_ = 1.39 V is applied to the LC, RCP is incident to the SAM selective chiral metasurface, and nearly all incident light (99.9%) is absorbed, so the metaholographic image has near-zero intensity (Figure 1C). In contrast, when *V*_ac_ = 1.18 V is applied to the LC, it transmits LCP, and the bright metaholographic images are reconstructed at the image plane (Figure 1C). The intensity of the holographic images can be continuously controlled by varying *V*_ac_ between 1.18 and 1.39 V. Various hologram intensities were experimentally achieved using 23 steps of polarization state (Figure 1C, Supplementary Material 1, Figure S1).

**Figure 2:**
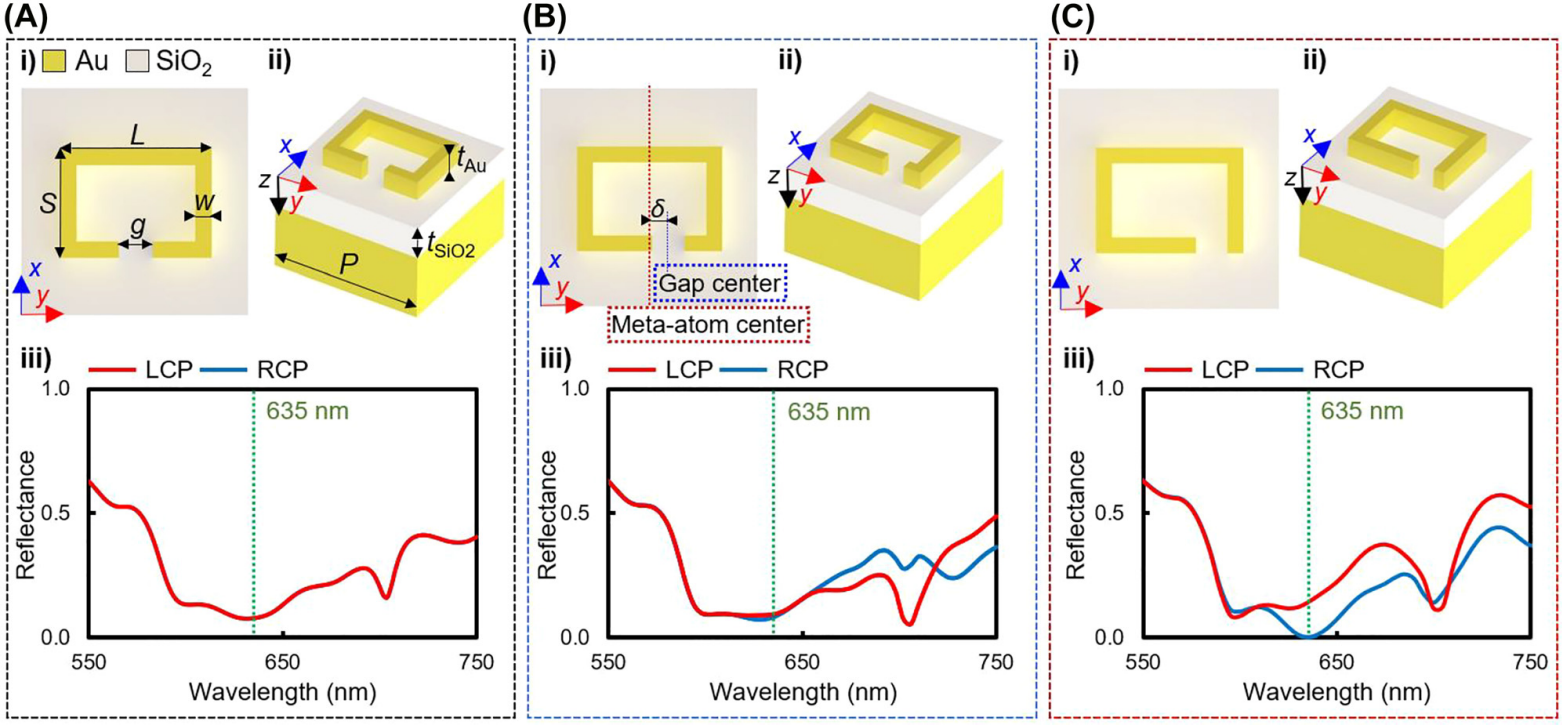
Effect of gap-shift on chiro-optical responses in split-ring resonators (SRR). Geometric parameters of the gap-shifted SRRs are long-axis length *L*, short-axis length *S*, gap *g*, gold width *w*, Au thickness *t*_Au_, and SiO_2_ thickness *t*_SiO2_, gap displacement *δ*, and periodicity *P*. Geometric parameters are *L* = 345 nm, *S* = 242 nm, *g* = 58 nm, *w* = 83 nm, *t*_Au_ = 57 nm, *t*_SiO2_ = 308 nm, and *p* = 450 nm. Simulated reflectance spectra of three SRRs, that have gaps in (A(i)) The center (*δ* = 0 nm) (B(i)) The mid-right (*δ* = 30 nm), and (C(i)) The right (*δ* = 60.5 nm). Red dotted line: center of unit cell; blue dotted line: center of gap. (i) Top-view, (ii) tilted view of unit cells, and (iii) simulated reflectance under LCP and RCP, respectively.

To achieve spin-selective chiral metasurfaces that perfectly absorb RCP at visible frequencies, gap-shifted split-ring resonators (SRR) are designed (Figure 2) using metal–dielectric–metal multilayer structures to produce strong gap-plasmonic responses [71] (Figure 2A(i)–C(i)). We simulate three SRRs with different gap displacements *δ* = 0, 30, and 60.5 nm, and with the same geometric parameter of length *L* = 345 nm of long axis, length *S* = 242 nm of short-axis, gap *g* = 58 nm, gold width *w* = 83 nm, gold thickness *t*_Au_ = 57 nm, SiO_2_ thickness *t*_SiO2_ = 308 nm, and periodicity *p* = 450 nm. The SRR with *δ* = 0 shows a non-chiroptical response due to symmetry, and therefore has the same reflectance under both RCP and LCP incident light (Figure 2A(iii)). However, for the gap-shifted SRRs (*δ* = 30, 60.5 nm), the displacement of the gap positions breaks the mirror symmetry, therefore, chiroptical properties are observed in that the reflectance of RCP and LCP incident light differ (Figure 2B(iii) and C(iii)). The circular dichroism in reflection (CDR) is defined as the ratio of the normalized difference of the total reflectance of incident LCP and RCP: (*R*_L_ − *R*_R_)/(*R*_L_ + *R*_R_), where *R* indicates reflectance, and the subscripts L and R represent the reflectance when LCP and RCP light is incident, respectively. The gap-shifted SRR with *δ* = 60.5 nm shows large inherent chiroptical characteristics, with a significant difference in reflectance under normal incident RCP and LCP illumination, achieving 99.9% absorption of RCP incidence, and 14.0% reflectance of LCP incident light at the wavelength *λ* = 635 nm (Figure 2C(iii)).

**Figure 3:**
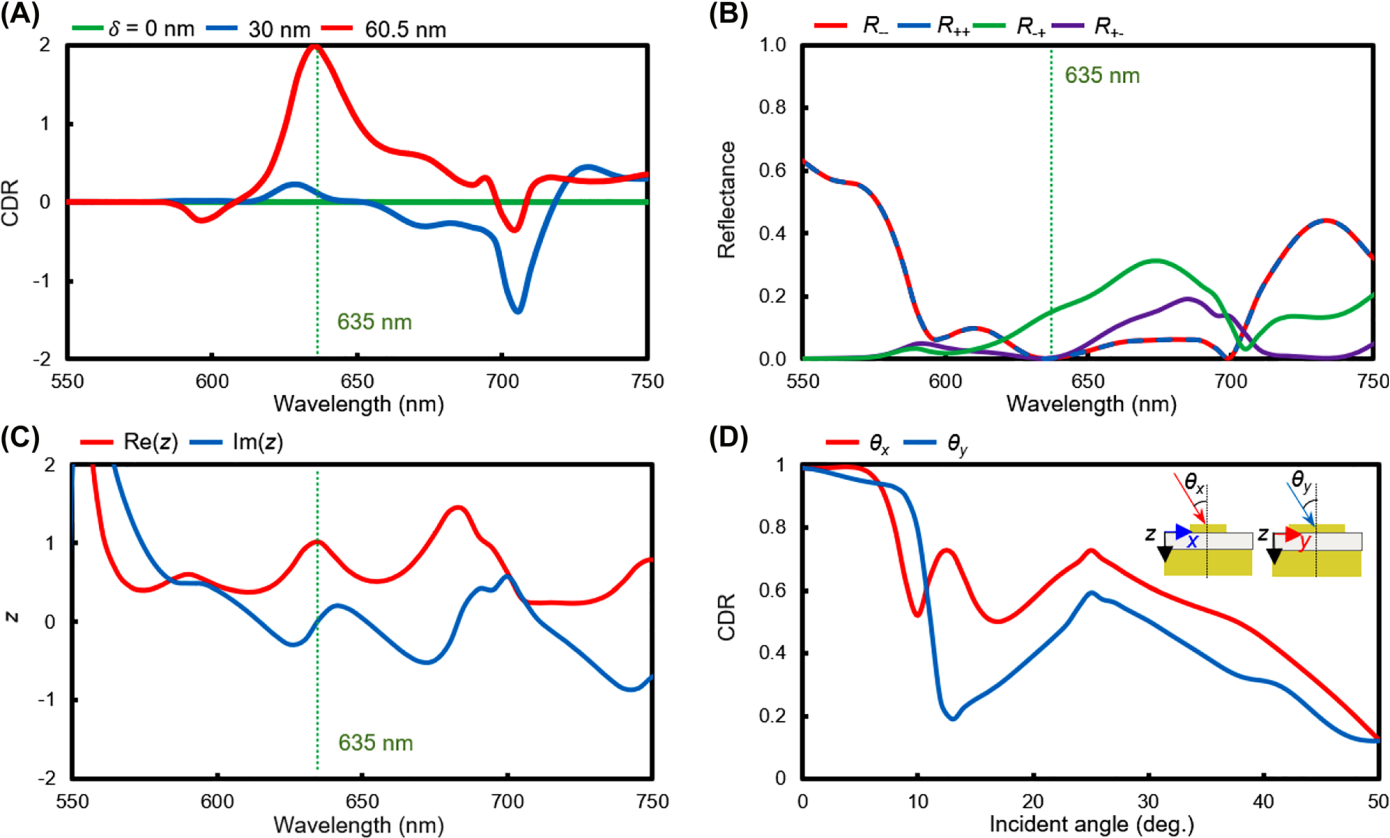
Chiroptical responses of the gap-shifted SRR. (A) Calculated circular dichroism in reflection (CDR) when the gap displacement *δ* is varied as 0, 30, and 60.5 nm. (B) Reflectance components of the gap-shifted SRR (*δ* = 60.5 nm). (C) Calculated effective impedance *z* of the gap-shifted SRR (*δ* = 60.5 nm) under RCP incidence. The dotted green line indicates the wavelength of 635 nm, where the metasurfaces perfectly absorb incident RCP. (D) Calculated CDR of the gap-shifted SRR (*δ* = 60.5 nm) depending on the incident angle from 0 to 50⁰. *θ*_*x*_ and *θ*_*y*_ denote incident angles with respect to the *yz-* and *xz-*plane, respectively.

The CDR spectra for the three SRRs (*δ* = 0, 30, and 60.5 nm) are simulated with commercial finite element methods (Multiphysics, COMSOL v5.6) (Figure 3A). At *δ* = 60.5 nm, the gap shifted-SRR exhibit strong CDR (0.99) at the wavelength of 635 nm. The SRR with *δ =* 0 nm is achiral, and therefore has zero CDR over the entire spectrum due to the symmetry of the geometry, and the SRRs with *δ =* 30 nm exhibit maximum CDR at the wavelength of 706 nm, but it cannot be applied to SAM selective metasurfaces due to low CDR (details about CDR of gap-shifted SRR with *δ =* 30 nm see Supplementary Material 2, Figure S2). To analyze SAM conversion, the LCP and RCP components (*R*_−−_, *R*_++_, *R*_−+_, and *R*_+−_) of reflectance are plotted in Figure 3B with subscripts + and − representing the LCP and RCP components, respectively; the former subscript indicates the output polarization states and the latter indicates the input. For example, *R*_+−_ denotes the reflectance component of the input RCP and output LCP. Thus, the total reflectance under RCP is the sum of *R*_+−_ and *R*_++_. For the gap-shifted SRR with *δ* *=* 60.5 nm, *R*_−+_ = 0.140 at *λ* = 635 nm and the other reflectance are near zero (*R*_+−_ = 5.90 × 10^–4^, *R*_++_ = 5.13 × 10^–5^, *R*_−−_ = 5.34 × 10^–5^), so RCP incident light is perfectly absorbed and only LCP incident light is reflected and converted to RCP light.

The impedance-matching conditions are further investigated to reveal the mechanism of perfect absorption in the gap-shifted SRR with *δ* = 60.5 nm. Assuming that the structure is homogeneous, the effective impedance *z* of an SRR is calculated using the *S*-parameter retrieval method [72]:

(1) $$z=\pm \sqrt{\frac{{\left(1+S_{11}\right)}^{2}-S_{21}^{2}}{{\left(1-S_{11}\right)}^{2}-S_{21}^{2}}}\text{,}$$

where *S* is the *S*-parameter element; and the former and latter subscripts represent the output and input components, respectively. When the impedance of the metasurface matches the air impedance, which is 1, the metasurface functions as a perfect absorber. The impedance of the gap-shifted SRR (*δ =* 60.5 nm) is calculated for RCP incidence (Figure 3C), where at *λ* = 635 nm, the real part of the impedance is 1.01, and the imaginary part is 0.0321, which are similar to the impedance of the air and there, yield perfect absorption. On the other hand, the effective impedance of *z* = 0.451 − 0.0190*i* under LCP incidence at *λ* = 635 nm does not match that of air, so perfect absorption is not achieved due to the impedance mismatch (Supplementary Material 3, Figure S2). In addition, the gap-shifted SRR (*δ* = 60.5 nm) showed robustness in incident angle. The CDR maintains >0.95 where an incident angle *θ*_*x*_ and *θ*_*y*_ are under 7⁰ and 6⁰, respectively. After that, they continuously decrease as the incident angle *θ*_*x*_ and *θ*_*y*_ increase to 50⁰ (Figure 3D, details in Supplementary Materials 4).

**Figure 4:**
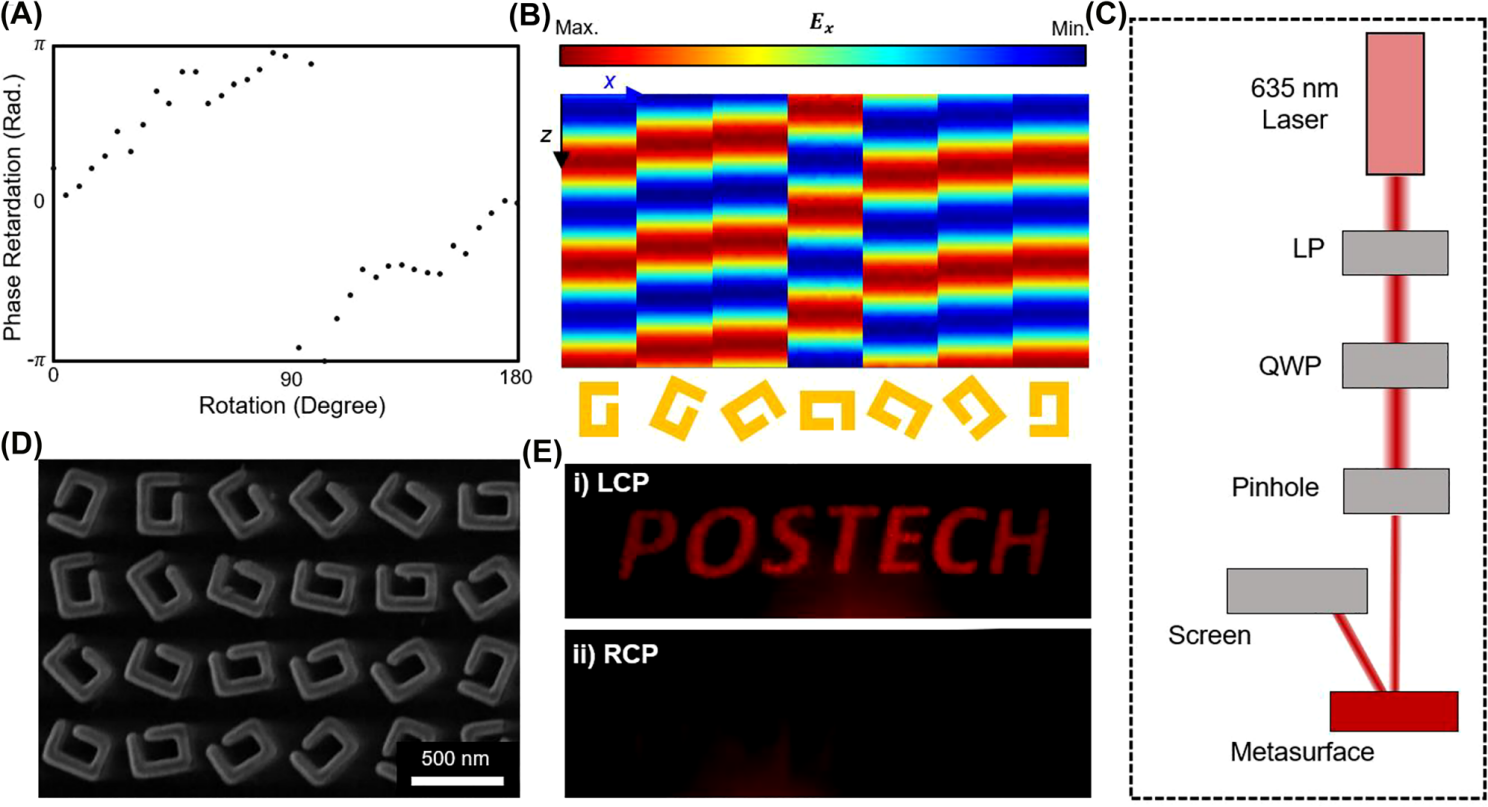
Demonstration of the spin-selective chiral metasurface. (A) Simulation of phase retardation depending on the rotation angle of the gap-shifted SRR under LCP. When LCP is incident to the metasurface, the phase of output light is retarded proportional to the rotation angle of gap-shifted SRRs. (B) Normalized electrical field ***E***_*x*_ of the reflected light from rotated gap-shifted SRR that shows the PB phase of the chiral nanostructure under incident LCP. (C) Optical setup designed with 635 nm laser, linear polarizer (LP), quarter-wave plate (QWP), and 300 μm diameter pinhole for the proof of concept of the spin-selective metahologram. Holographic metasurface was designed as an off-axis hologram to avoid overlapping of zeroth-order diffraction light and hologram image. (D) Top view scanning electron microscope image of the fabricated metasurface. (E) Holographic image ‘POSTECH’ is obtained under (i) incident LCP, and disappears under (ii) RCP. Twin images are not obtained under both RCP and LCP, which are omitted from the figure for clarity.

For the spin-selective metahologram that works at *λ* = 635 nm, the concept of reflective geometric metasurfaces [73] is used to demonstrate metaholography by using the gap-shifted SRR (*δ* = 60.5 nm) as a unit cell, because the SRR completely absorbs RCP and converts 14.0% of LCP to RCP, which is essential for the use of Pancharatnam–Berry (PB) phase, as PB phase utilizes conversion efficiency to modulate the phase. To reconstruct the holographic images, the phase distribution of the hologram is retrieved using the iterative Gerchberg–Saxton (GS) algorithm (details in Supplementary Material 5). The phase of each pixel is encoded using the geometric PB phase, which achieves phase 2*θ* by rotating the nanostructure unit cells by an angle *θ*, and was designed to exhibit off-axis images to avoid overlapping with zeroth-order diffraction. By rotating the gap-shifted SRR (*δ* = 60.5 nm), from 0 to 180° in the *xy*-plane, 0 to 2*π* phase is achieved by the PB phase as shown in Figure 4A. The phase retardation is almost proportional to the rotation angle of the SRR, and small variations may be a result of interaction between adjacent SRRs. Reflected electric-field profiles are calculated when the SRR is rotated in eight steps between 0 and 180°; the profiles shift in proportion to the rotation (Figure 4B).

As a proof-of-concept of the spin-selective metahologram, holographic images are measured using a homemade optical setup that is composed of a compact diode 635 nm laser, linear polarizer (LP), quarter-wave plates (QWP), and pinhole with a diameter of 300 µm (Figure 4C). LCP and RCP are created by placing the LP and the QWP on the optical paths. The light is incident on the fabricated reflective spin-selective metasurface composed of rotated gap-shifted SRR (*δ* = 60.5 nm) unit cells that are rotated to encode the desired phase at each spatial location, and the screen was placed off-axis at the same side of the beam to avoid the zeroth-order beam. Figure 4D shows an SEM image of part of the fabricated spin-selective metasurface. Using the experimental setup in Figure 4C, a clear holographic image of the word ‘POSTECH’ is observed on the image plane when illuminated by LCP (Figure 4E(i)); however, the image disappears under RCP incidence and also with the zeroth-order diffracted light, because the metasurface completely absorbs RCP (Figure 4E(ii)). This result demonstrates the spin-selective characteristics of the metasurface.

**Figure 5:**
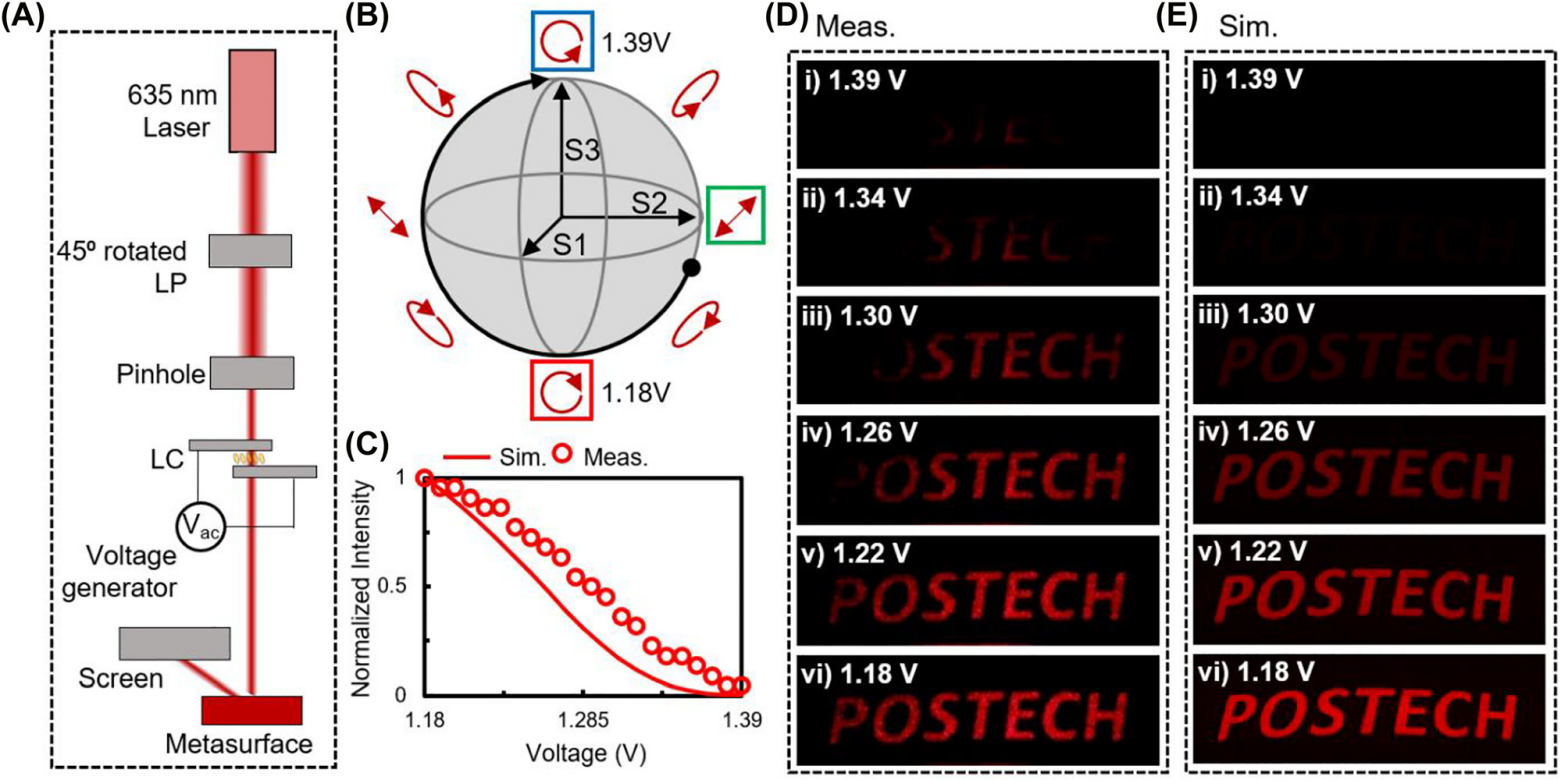
Demonstration of electrically-tunable intensity using LC-assisted spin-selective chiral metasurfaces. (A) Schematic of the optical setup for electrically-tunable intensity with LC-assisted spin-selective chiral metasurfaces. (B) Output polarization states pass through the LC when 45° rotated linearly polarized light is incident. Green, red, and blue squares: Incident and output polarization states when *V*_ac_ is 1.18 and 1.39 V, respectively. Black dot: polarization states at *V*_ac_ = 0 V (C) Calculated (solid line) and measured (dotted line) intensity of metaholograms. (D) Measured and (E) simulated continuous intensity-tunable holograms when *V*_ac_ were (i) 1.39 V, (ii) 1.34 V, (iii) 1.30 V, (iv) 1.26 V, (v) 1.22 V, and (vi) 1.18 V.

To realize a continuously-tunable intensity of hologram, the gap-shifted SRRs were integrated with LCs, which act as a retarder and function as an electrically-tunable QWP (Figure 5A). The LCs, 4-cyano-4′pentylbiphenyl (5CB); their refractive index (extraordinary refractive index *n*_e_) on the long axis differs from that on the short axis (ordinary refractive index *n*_o_). The effective refractive index of the LC cells *n*_eff_ is defined as $n_{\text{o}}n_{\text{e}}/\sqrt{n_{\text{o}}^{2}{\cos\;}^{2}\left(\alpha \right)+n_{\text{e}}^{2}{\cos\;}^{2}\left(\alpha \right)}-n_{\text{o}}$ , where *α* is the angle between the rubbing direction and the long axis [74, 75]. The LCs reorder their long axis to be parallel to the direction of an applied electrical field, varying their optical axis in response to *V*_ac_, allowing them to act as phase retarders. The amount of the phase retardation is defined as $\tau =\int_{0}^{t}2\pi \Delta n_{\text{eff}}\left(z\right)/\lambda \text{d}z$ rad, where *t* is the thickness of the LC cell [74, 75]. Consequently, by combining the LC with the 45°-rotated LP, the output light can be controlled from LCP to RCP by precisely manipulating *V*_ac_. The possible polarization states of output light can be represented using a Poincaré sphere (Figure 5B). The polarization is rotated with respect to the S1 axis of the Poincaré sphere when the input light is 45°-rotated linearly polarized light (Figure 5B, green square). A detailed description of LC for implanting in metasurfaces is given in refs. [65, 76].

For the case when 45°-rotated linearly polarized light (green square in S2–S3 plane on the Poincaré sphere) is incident to the LC, when *V*_ac_ = 0 V, the LC had *τ* = 18.3 rad, which is 2.9 turns on the S2–S3 plane in Poincaré sphere with respect to the S1 axis (Figure 5B, black dot). When *V*_ac_ is applied to the LC, the *τ* of the LC decreases, and the polarization states that pass through the LC rotates clockwise on the S2–S3 plane in the Poincaré sphere with respect to the S1 axis (Figure 5B) [65]. Here, the S1 axis corresponds to the rubbing direction. By continuously increasing *V*_ac_, we determined that *V*_ac_ = 1.39 V results in RCP (blue square) output polarization, and *V*_ac_ = 1.18 V results in LCP (red square) output polarization, respectively. *V*_ac_ between these RCP and LCP values creates elliptical polarization states that are composed of RCP and LCP, and when *V*_ac_ increases and get closer to 1.39 V, the LCP ratio decreases so the intensity of the hologram decreases.

The intensity of the reconstructed hologram image was measured, and it is consistent with the simulated conversion efficiency of the gap-shifted SRR (Figure 5C). The simulation was conducted by changing the input polarization states to the spin-selective metasurface that corresponds to applied *V*_ac_; the contrast of reconstructed hologram intensity reaches ∼1:240, which agrees with the calculated conversion efficiency (14.0% for LCP incidence, 0.0590% for RCP incidence). The reconstructed hologram intensity is measured as 0.022 μW at *V*_ac_ = 1.18 V and 0.001 μW at *V*_ac_ = 1.39 V, which is 22 times larger, reaching CDR of 0.91 (details in Section 4). The discrepancy between simulated and measured intensity originates from (1) minor thickness variation in homemade LC cells, and (2) fabrication defects in realized metasurfaces. These discrepancies can be corrected through optimization of the LC-made and the metasurface manufacturing process. As a result, by continuously modulating *V*_ac_ (in the experiment, increments of 0.01 V) from 1.39 to 1.18 V, the intensity of the holographic image ‘POSTECH’ is continuously increased (Figures 5D and S3). We assume that the incident light has uniform light intensity distribution (not the Gaussian beam) because the beam size is enough to fully cover that of metasurfaces.

To verify the intensity-tunable metahologram, measured holographic images are compared with simulated reconstructed holographic images depending on applied *V*_ac_ from 1.39 to 1.18 V (Figure 5E). In the measured reconstructed holograms, the center characters of the word ‘ST’ exhibit higher intensity than that of the side ones. However, simulated reconstructed holograms have coherent intensity tunability regardless of the position of the characters. The different intensity may be induced by different distances from the reflective metasurface to the point of the reconstructed image on the screen. Side characters have more distance than center characters, so they are turned off more rapidly. This discrepancy can be further corrected by modifying the intensity of the initial target images.

## 3 Conclusions

In conclusion, we designed spin-selective chiral metasurfaces composed of the gap-shifted SRR and implanted them with LC cells to achieve electrically intensity-tunable metaholography. The measured CDR of our device approaches 0.91, and its theoretical value reaches 0.99 at *λ* = 635 nm, enabling perfect SAM selection by the gap-shifted SRR. The measured CDR of 0.91 is the highest value of any previously reported PB-phase based chiral plasmonic metasurfaces working at the visible and infrared regions (Table 1). In addition, the SAM selective chiral metasurfaces can be fabricated using conventional electron-beam lithography and deposition processes, so it has higher fabrication feasibility than previously-reported three-dimensional chiral plasmonic metasurfaces such as helical structures [66] (Table 1).

**Table 1:** Comparison of chiral structural materials for PB-phase metasurfaces working at the near-infrared and visible frequencies. All CDR value is calculated as an absolute value of (*R*_R_ − *R*_L_)/(*R*_R_ + *R*_L_) for clear comparison. For transmissive metasurfaces, CDRs are replaced by circular dichroism on transmission (CDT) that is expressed as an absolute value of (*T*_R_ − *T*_L_)/(*T*_R_ + *T*_L_). Superscripts: CDR, CDT calculated by (a): simulation, (b): measurement.

Year [Reference]	Wavelength (nm)	CDR, CDT	Materials	Fabrication feasibility (Geometric dimension)	Usage
Our work	635	0.99^a^, 0.91^b^	Au, SiO_2_	High (2D)	Hologram
2017 [79]	1,560	0.99^a^, 0.78^b^	Si	High (2D)	Wave plate
2018 [80]	1,655	0.86^a^	Ge	High (2D)	Hologram
2018 [66]	806	0.91^a^, 0.77^b^	Au	Low (3D)	Hologram
2020 [81]	792	0.30^a^	Lithium niobate	High (2D)	–
2020 [82]	633	0.43^a^, 0.45^b^	a-Si:H	High (2D)	Hologram
2020 [83]	1,384^a^, 1,400^b^	0.99^a^, 0.88^b^	Al, SiO_2_	High (2D)	Color printing
2021 [34]	633	0.82^a^, 0.84^b^	a-Si:H	High (2D)	–

Compared to dielectric metasurfaces that exploit the PB phase, the SAM selective chiral metasurface has the advantage that the output phase map is not reversed when the input handedness is converted. When input light has imperfect circular polarization states such as elliptical or linear polarization, the conventional dielectric metasurface for metaholograms and metalenses cannot avoid twin-holographic images and dispersion of light, respectively. Although combining the geometrical phase with the propagation phase has been evaluated as a way to generate independent responses depending on the handedness of input circularly polarized light [77, 78], their structured materials have low fabrication tolerance, because they require both desired amplitude and phase profiles. Thus, the SAM selective metasurfaces can be beneficial to avoid noise (e.g., twin hologram images) caused by imperfectly polarized light when metalenses and metaholograms are designed.

Although previous research commented that plasmonic structures have the limitation on their unavoidable intrinsic losses and that high index dielectrics (e.g. titanium dioxide, silicon nitride, etc.) will be the dominant material, we believe that their optical losses can be used in a practical way (herein, perfect absorption of certain circularly polarized light), opening new application fields of plasmonic metasurfaces. Considering that LC cells can be closely packed by locating them directly on the metasurfaces to make a more compact form factor [65, 76], we believe that the SAM selective chiral metasurface will become one more option for photonic devices that work at visible frequencies.

## 4 Experimental section

### 4.1 Numerical simulations

Numerical simulations were conducted using the commercially available finite element method (FEM) solver, COMSOL Multiphysics v5.6. Simulations of reflectance coefficient, impedance, phases, and reflected intensity profiles were calculated using periodic boundary conditions in the *x* and *y* directions, and perfect boundary conditions in the *z*-direction.

### 4.2 Fabrication of metasurfaces

The designed metasurfaces were fabricated using conventional manufacturing methods, including electron-beam lithography and electron beam deposition. Firstly, a 300 nm-thick gold (Au) and 308 nm-thick silicon dioxide (SiO_2_) layer were sequentially deposited on a silicon (Si) wafer using electron beam deposition. Electron-beam resists (Microchem, PMMA 495 A6) were spin-coated on the sample at 2000 rpm for 1 min, then baked at 180 °C for 5 min. The resist was exposed to electron-beam following prepared nanopatterns at 100 kV using electron-beam lithography (Elionix, ELS-7000). And then, the sample was immersed in a developer (Microchem, MIBK: IPA = 1:3) at 0 °C for 10 min to remove exposed patterns. Electron beam deposition was used to deposit a 57 nm-thick Au film on the sample, and it was immersed in acetone at 60 °C for 12 h to lift off the unexposed area to complete the sample fabrication.

### 4.3 Fabrication of LC cells

The LC cells were prepared using polyimides (Nissan Chemical Korea) for the alignment layer, and indium tin oxide (ITO) coated glass plates. The polyimides were spin-coated, baked, and rubbed to create a rubbing direction for unidirectional alignment for LCs. Two substrates were prepared using the same method as in Section 4.2. After assembling two substrates with a gap of 10 µm by using UV-glue (Norland Products Inc., NOA 65) and glass spacer, sandwiched cells were filled with nematic LCs (Jiangsu Hucheng Display Technology Co., Ltd). Specific methods are detained in refs. [65, 76].

## Supplementary Material

Supplementary Material
