# Supplementary material for: Gap-plasmon-driven spin angular momentum selection of chiral metasurfaces for intensity-tunable metaholography working at visible frequencies
Source: Nanophotonics. 2022 May 16;11(17):4123–33. doi: 10.1515/nanoph-2022-0075 (PMC11501853; doi:10.1515/nanoph-2022-0075)
Supplement: Supplementary file 1 — Supplementary Material [file j_nanoph-2022-0075_suppl.docx]

Supplementary Information for

**Gap-plasmon-driven spin angular momentum selection of chiral metasurfaces for intensity-tunable metaholography working at visible frequencies**

Younghwan Yang^a^, Hongyoon Kim^a^, Trevon Badloe, and Junsuk Rho^*^

^a^**Younghwan Yang** and **Hongyoon Kim**: These authors contributed equally to this work.

^*^**Corresponding authors**: **Junsuk Rho**, Department of Mechanical Engineering, POSTECH, Pohang 37673, Republic of Korea; Department of Chemical Engineering, POSTECH, Pohang 37673, Republic of Korea; POSCO-POSCTECH-RIST Convergence Research Center for Flat Optics and Metaphotonics, Pohang 37673, Republic of Korea; and National Institute of Nanomaterials Technology (NINT), Pohang 37673, Republic of Korea, E-mail: [jsrho@postech.ac.kr](mailto:jsrho@postech.ac.kr), https://orcid.org/0000- 0002-2179-2890

**Younghwan Yang**, **Hongyoon Kim**, and **Trevon Badloe**: Department of Mechanical Engineering, Pohang University of Science and Technology (POSTECH), Pohang 37673, Republic of Korea. https://orcid.org/0000-0003-2173-4217 (Y. Yang), https://orcid.org/0000-0003-2113-3921 (H. Kim)

Supplementary Material 1: Tunable hologram intensity with 23-levels

In the experiment, 23 steps of tunable intensity are demonstrated by manipulating *V*_ac_ from 1.18 to 1.40 V in increments of 0.01 V (Figure S1). Intensities gradually decreased as *V*_ac_ was increased.

| 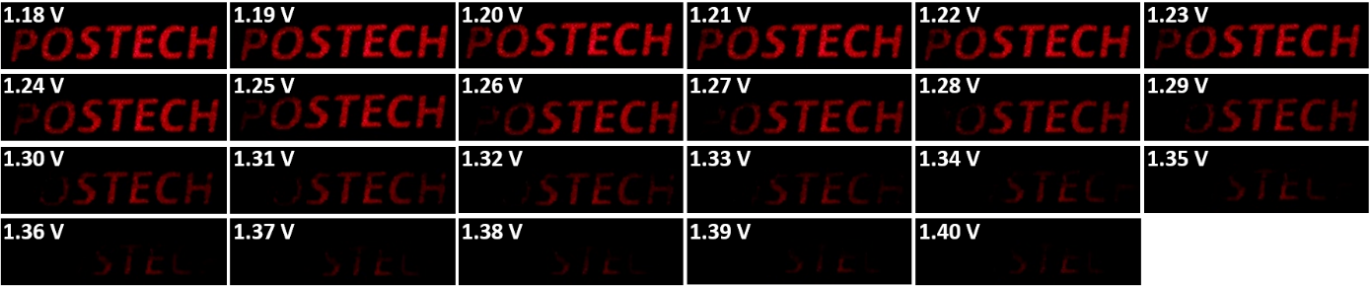 |
| --- |
| Figure S1: Captured image of intensity tunable metahologram. Different intensities are measured using LC-integrated SAM selective chiral metasurfaces. Voltage applied to the LC was adjusted from 1.18 to 1.40 V, and the intensity decreased as voltage increased. |

Supplementary Material 2: Chiroptical responses of gap-shifted SRR with *δ* = 30 nm at *λ* = 706 nm

The gap-shifted SRR with *δ =* 30 nm exhibit CDRs with a value of 0.69 at the wavelength of 706 nm. To analyze CDR at *δ =* 30 nm, the charge distribution and current density are simulated under different circularly polarized light at the wavelength of 550 and 706 nm, where CDRs are 5.2 × 10^-5^ and 0.69, respectively. The charge distributions show that the gap-shifted SRR exhibit strong free electron oscillation (red arrow in **Figure R1**), and induced significant positive charge is observed at the upper and left side under LCP incident (color bar in **Figure R1**). The strong free electron oscillation can induce different light-matter interactions between incident LCP and RCP, achieving a high CDR.

| 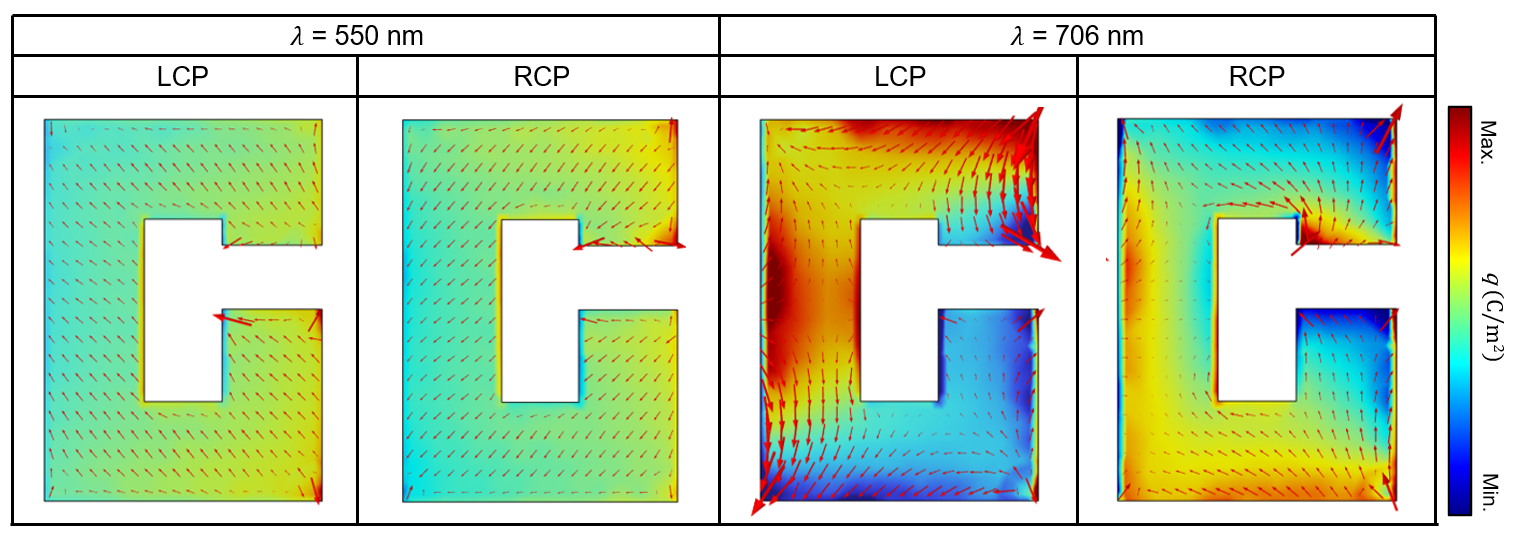 |
| --- |
| Figure S2: Calculated surface charge distribution (*q*) and current density with gap-shifted SRR (*δ* = 30 nm). Under LCP and RCP at the wavelength of 550 and 706 nm, different charge distribution and current density is observed. The different profile of free-electron oscillation induces different reflection intensity of light, where CDR of 0.69 is observed. |

Supplementary Material 3: Impedance of incident LCP

The impedance of the gap-shifted SRR (*δ =* 60.5 nm) with LCP incidence is demonstrated (**Figure S2**). Compared with the impedance of RCP, the real part of the effective impedance *z* is 0.864, and the imaginary part is 0.861, which does not match the air impedance.

| 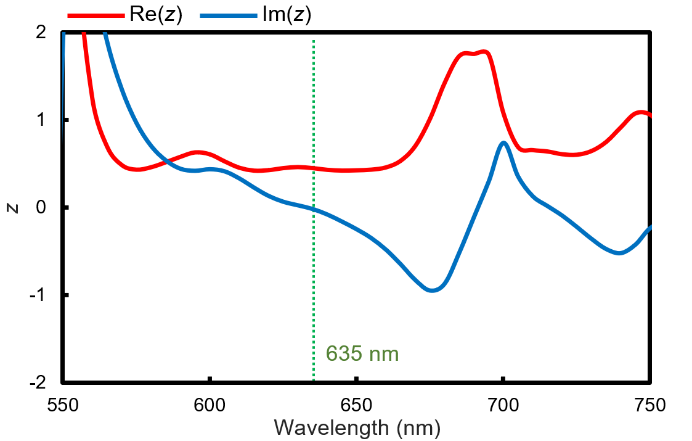 |
| --- |
| Figure S3: Calculated effective impedance *z* of the gap-shifted SRR (*δ =* 60.5 nm) with LCP incidence. Green dotted line: *λ* = 635 nm. At * * = 635 nm, real *z* = 0.451 and imag *z* = -0.190. |

Supplementary Material 4: Effect of incident angle on CDR

To investigate the effect of the incident angle on CDR, reflected RCP and LCP with the gap-shifted SRR with *δ* = 60.5 nm are calculated by varying incident angle from 0 to 50⁰. At normal incidence, the reflectance for LCP incidence is 14.0% and the reflectance for RCP incidence is 0.1%. and for incident angle $\theta_{x}, \theta_{y}$ < 7˚, the RCP reflectance maintains under 1%, and the LCP reflectance retains 14.0% indicating the incident angle robustness of the proposed gap-shifted SRR. The difference of LCP and RCP continuously decreases from 7 to 50˚, decreasing the chiroptical responses of gap-shifted SRR.

| 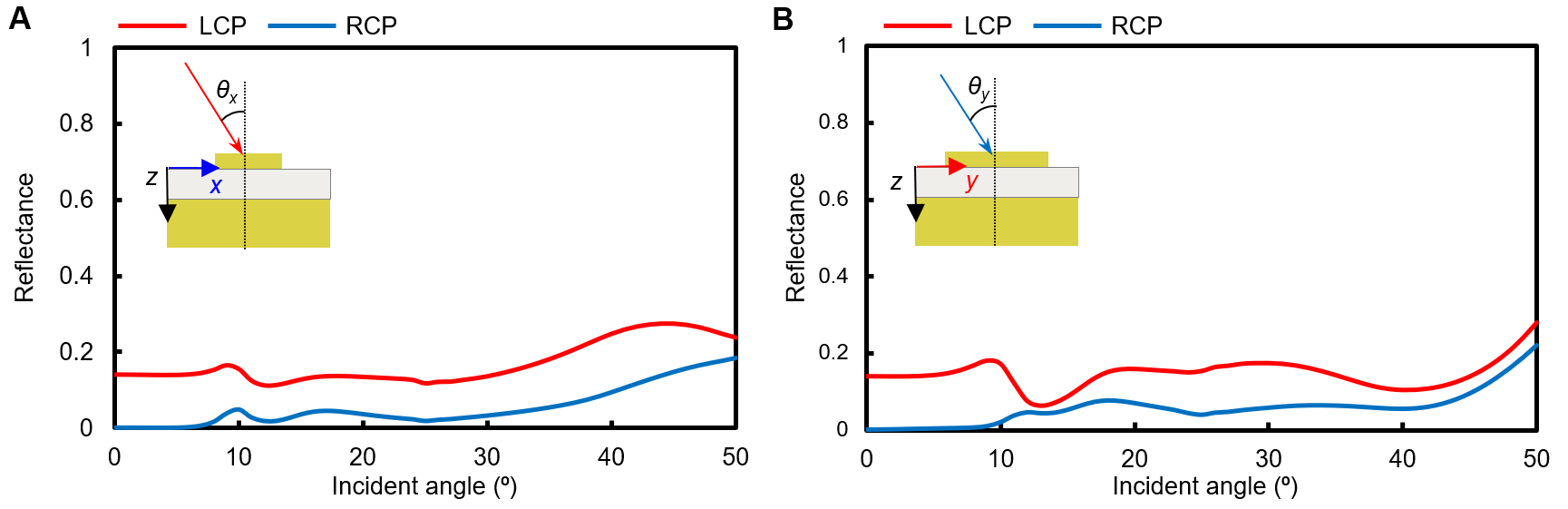 |
| --- |
| Figure S4: Calculated reflection of LCP and RCP incidence depending on an incident angle. Reflectance depending on (A) *θ_x_* and (B) *θ_y_*. |

Supplementary Material 5: Computer-generated hologram with GS-algorithm

Using 2π coverage of the gap-shifted SRR structures, a typical GS-algorithm is employed to generate a phase map of a phase-only hologram. The GS-algorithm creates a phase map with iterative Fourier transformation to minimize errors between target and generated images (Figure S5). To reduce pincushion distortion, the target image is compensated, and it makes slightly different images between the target and generated image (Figure S5A, and Figure S5B).

| 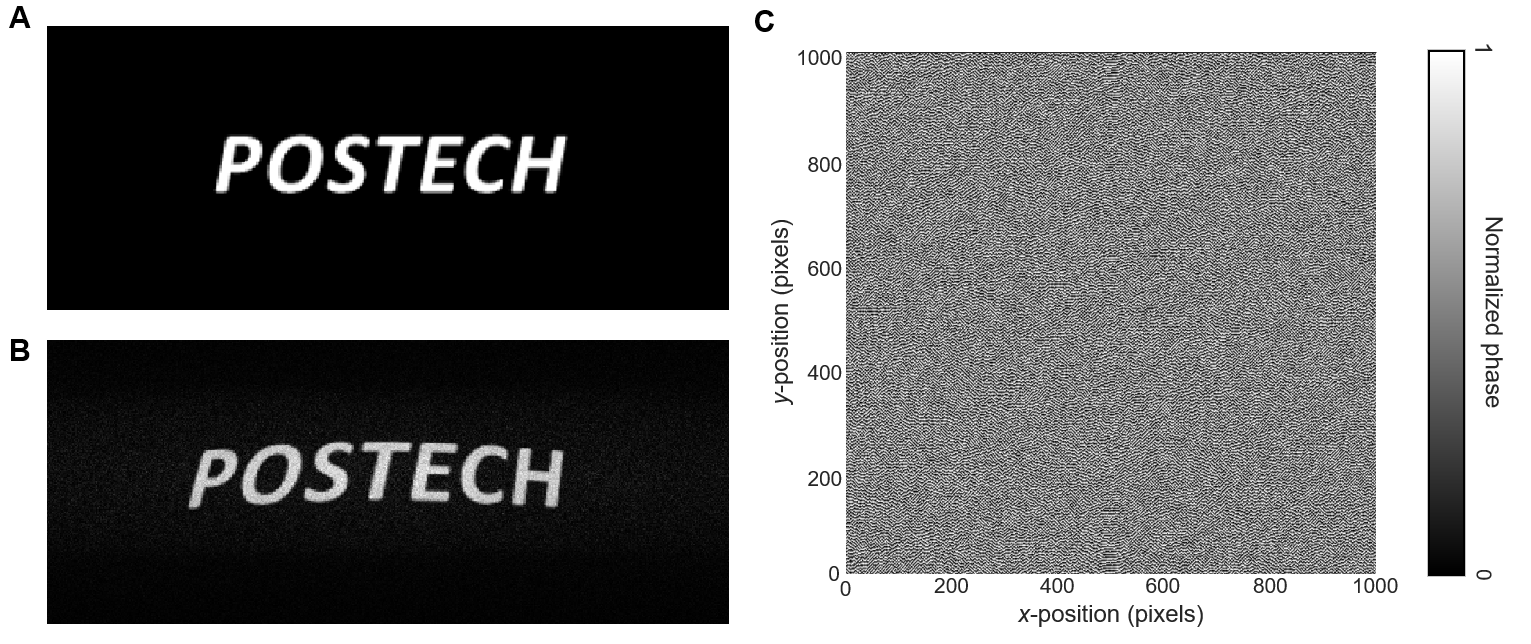 |
| --- |
| Figure S5: Target and generated computer-generated hologram images. GS-algorithm is used for creating a phase-only computer-generated hologram. (A) Target images, ‘POSTECH’ is used, and (B) generated images are calculated. Generated images are compensated to prevent pincushion distortion. (C) Local phase retardation is achieved with 1000-by-1000 pixels metasurfaces with a calculated phase map. |
